# Recurrent infarcts from thyroid cartilage compression of an aberrant vertebral artery: rare, easily overlooked, but treatable

**DOI:** 10.1007/s00415-023-11896-8

**Published:** 2023-08-07

**Authors:** K. W. Sühs, W. Koestner, M. Schütze, P. Bronzlik, E. J. Hermann, M. Durisin, M. Polemikos, J. K. Krauss, G. U. Höglinger

**Affiliations:** 1 Department of Neurology, Hannover Medical School, Carl-Neuberg-Straße 1, 30625 Hannover, Germany; 2 Department of Radiology and Neuroradiology, St. Bernward Hospital, Treibestraße 9, 31134 Hildesheim, Germany; 3 Department of Neuroradiology, Hannover Medical School, Hannover, Germany; 4 Department of Neurosurgery, Hannover Medical School, Hannover, Germany; 5 Department of Otolaryngology, Otto-Von-Guericke-University Magdeburg, Magdeburg, Germany; 6 Department of Neurology, Ludwig-Maximilians-University, Munich, Germany

## Case 1

**Fig. 1:**
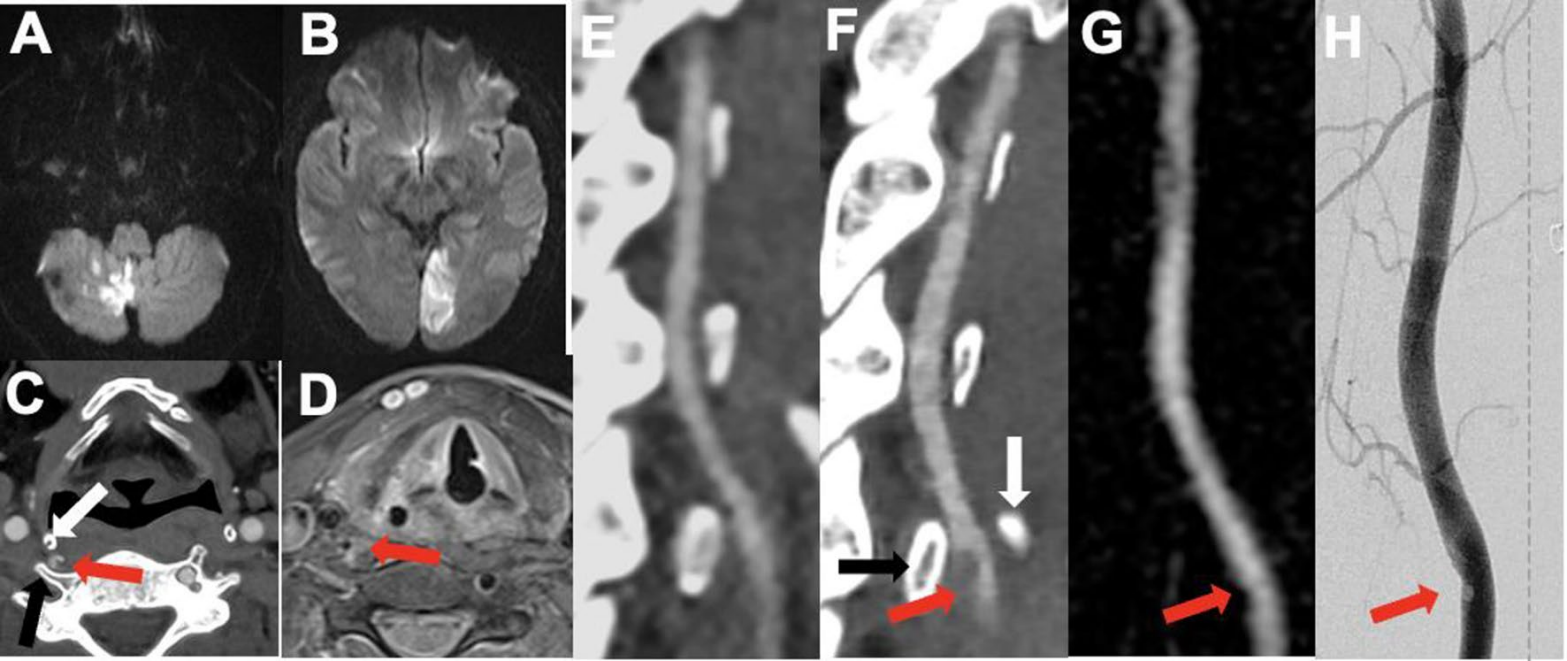
Brain MRI diffusion-weighted images depict right PICA infarct on d0 (**A**) and left PCA infarct on d25 (**B**). **C** CT angiogram shows compression of aVA-C4 at d25 between the superior cornu of the thyroid cartilage (white arrow) and the C5 transverse process (black arrow). A crescent-filling defect is depicted with a red arrow. **D** No signal abnormalities were observed in fat-saturated T2w sequences at d30. A red arrow depicts aVA-C4. **E** CT angiogram on d0 does not show luminal irregularity. **F** Corresponding sagittal reformation to the axial images depicted in (**C**). On day 30, the filling defect was reduced on MR angiography (**G**) and in digital subtraction angiogram (**H**)

A 36-year-old man presented with vertigo, headache, and unsteady gait. On examination, we observed hypermetric vertical saccades, and truncal ataxia. MRI revealed acute bilateral and subacute right cerebellar infarctions (Fig. 1A) and chronic infarctions in both cerebellar hemispheres and in the left thalamus. However, CTA was unremarkable, except for an aberrant right vertebral artery (VA) entering the C4 foramen transversarium (aVA-C4). Laboratory examinations, a whole-body FDG-PET-CT scan, and cardiac assessments were unremarkable. The patient was put on acetylsalicylic acid (ASS) and discharged. Twenty-five days later, he presented with a homonymous hemianopsia to the right and MRI revealed acute left and subacute right PCA infarction (Fig. 1B). In contrast to the first CTA, the superior cornu of the thyroid cartilage was in close proximity to aVA-C4 anterior to the C5 transverse process (Fig. 1C, F). In addition, a crescent-filling defect was noted at the site of vascular injury (Fig. 1C, F). Three and 5 days later, the patient suffered new infarcts in the right PICA, AICA, and SCA territories (Table 1). The luminal filling defect was reduced on MRA and DSA (Fig. 1G, H), documenting thrombus release. Notably, fat-saturated T2w sequences did not show vessel wall abnormalities (Fig. 1D). In total, six different acute/subacute infarctions in the right VA-territory were documented within 30 days (Table 1). Therefore, we decided to reduce mechanical stress from aVA-C4 by resection of the anterior tubercle of the C5 transverse process and the superior cornu of the thyroid cartilage. The resections were performed by a neurosurgery and ENT team. The patient was put on dabigatran for 6 months without experiencing further strokes (currently, since 22 months).

**Table 1:** Evaluation of vascular territories and infarct size

	Acute infarction	Subacute infarction	No. of new infarcts	Max. infarct size [cm^2^]	Treatment	No. of vascular events
Case 1						
d0	R SCA, R PICA, L PCA	R SCA	7	3.0	ASS	2
d25	L PCA	R PCA	2	7.1	ASS	4
d28	R PICA, R AICA	–	2	2.0	ASS	5
d30	R SCA	–	8	11.3	ASS	6
d36	–	–			Surgery, dabigatran	
Case 2						
d0	R thalamus, R SCA, R PICA	R AICA	9	0.07	ASS	2
d15	L thalamus, L SCA, R PICA	L AICA	5	0.04	ASS	4
d32	–	L SCA	1	0.04	ASS	5
d84	–	L AICA	1	0.03	Dabigatran	6
d89	L SCA	–	2	0.02	Dabigatran	7
d92	L SCA, L AICA, R PICA, R AICA	–	8	0.02	Dabigatran and cortisone	8
d96	R SCA	–	3	0.05	Dabigatran and cortisone	9
d118	R AICA, R PICA, L AICA	–	3	17.2	Dabigatran and cortisone	10
d121	L pons	–	1	0.08	Dabigatran and cortisone	11
d129					Surgery, phenprocoumon, ASS	

For detection of infarcts DWI-MRI was used, expect for d84, d89, and d118 in Case 2 CT was used. The max. infarct area on a single axial DWI-image is depicted

*SCA* superior cerebellar artery, *PICA* posterior inferior cerebellar artery, *AICA* anterior inferior cerebellar artery, *PCA* posterior cerebral artery

## Case 2

**Fig. 2:**
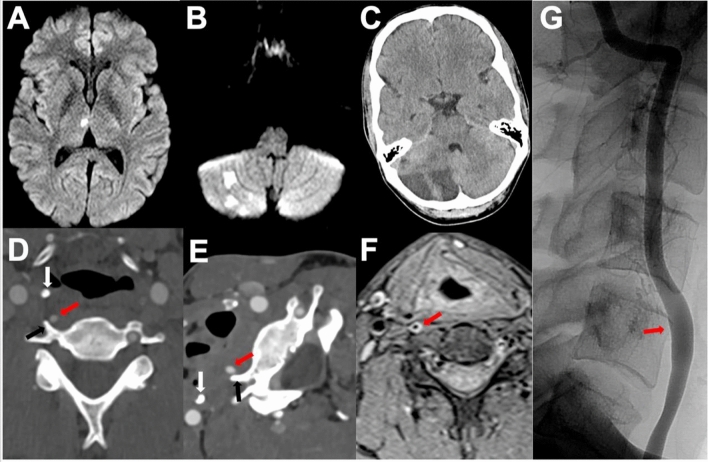
(A, B) Diffusion-weighted sequences depict right thalamic infarction (**A**) and right PICA infarcts (**B**) at d0. **C** Right-sided SCA infarct was observed in computed tomography on d118. No luminal irregularities were detected in aVA-C4 (red arrow) at d15 without (**D**) and with neck rotation to the right (**E**). **F** Fat-saturated T1w-sequences are depicted at d40. T1w hyperintensity of aVA-C4 is labeled with a red arrow. **G** DSA of aVA-C4 depicts minimal dilatation of aVA-C4 proximal to the entrance in the foramen transversarium C4 at d38

A 28-year-old man presented with headache, vertigo, and nausea. He reported recurrent episodes of vertigo in the last months. On examination, he had a tendency to fall to the left. MRI revealed acute right thalamic infarction and acute/subacute right cerebellar infarctions (Fig. 2A, B). The patient’s medical history included a focal epilepsy under antiepileptic treatment. A heterozygous factor V Leiden mutation was detected. Cardiologic diagnostics, CSF examination, FDG-PET/CT, and laboratory tests for vasculitis/autoimmune encephalitis were unremarkable. CTA revealed a right-sided aVA-C4 without luminal abnormalities. In addition, the left VA entered at the C5 level (aVA-C5). Dynamic imaging in different head positions with duplex sonography, CTA, and DSA did not show VA compression (Fig. 2E and data not shown). MRI revealed a subtle circular T1w hyperintensity of the vessel wall of aVA-C4 anterior to of the C5 transvers process (Fig. 2E), whereas T2w sequences were normal. A minimal fusiform dilatation of aVA-C4 in the same position was also noted in MRA, CTA, and DSA (Fig. 2G). After three further infarcts, treatment was switched from ASS to dabigatran; however, he suffered two more infarcts (Table 1). Assuming intracranial vasculitis as differential diagnosis the patient was treated with prednisolone in addition to dabigatran. Nevertheless, new infarcts occurred in the following weeks (Table 1). Notably, the sizes of these infarcts were very small with a maximum size of below 0.1 cm^2^ on axial DWI sequences (Table 1). Finally, MRI revealed large right-sided PICA and AICA infarcts (Fig. 1C, Table 1). In total, the patient had 11 infarcts within 17 weeks in the right VA territory (Table 1). Thus, we considered a vessel injury that is undetectable with luminal imaging. Consequently, as ultima ratio, the same surgical procedure as in patient 1 was performed. The patient was put on phenprocoumon and ASS for 6 months followed by ASS monotherapy without experiencing further strokes (currently, since 11 months).

## Discussion

Compression of an aberrant VA entering at the C3, C4, or C5 level (aVA-C3-5) is a rare cause of recurrent infarcts in the posterior circulation [1–5]. To the best of our knowledge, only six previous studies have described thyroid cartilage compression as the cause [6–11]. Dabus et al. reported on compression of aVA-C4 in front of the C6 transverse process [7]. Karle et al. demonstrated compression of aVA-C3 with neck flexion [6]. Another study reported aVA-C4 compression with neck rotation and extension [9]. Haertel et al. presented aVA-C4 compression related to neck rotation and swallowing [12]. Thus, the exact position of VA compression might depend on individual factors. Laryngoplasty as treatment of cartilage compression was introduced by Karle et al. [6]. Recently, four other studies were published [8–10, 12]. Spence et al. reported successful treatment in a patient with a right-sided aVA-C5 and a left-sided aVA-C3 [8]. Furthermore, a case of Bow Hunter’s syndrome from aVA-C4 compression was treated with laryngoplasty [9]. Haertel et al. presented two cases [12]. In the first case, stenting of aVA-C3 was complicated by stent-fracture and was treated with surgical bypass afterward [12]. In the second patient, aVA-C4 compression was successfully treated with laryngoplasty [12].

Imaging diagnostics of cartilage compression presents several challenges: awareness of the aberrant vessel course, finding the compressive head position, and detection of vessel injury. In our first case, we could demonstrate aVA-C4 compression by the superior cornu. However, in the initial CTA, the proximity of the superior cornu to aVA-C4 was not observed. In patient 2, we did not find VA impingement on neck rotation or flexion/extension (Fig. 2E). Nevertheless, during surgery, the laryngeal compression was obvious in both cases. All previous studies used luminal filling defects in CTA as a proof of dissection. We also found a luminal filling defect in patient 1, accounting for an appositional thrombus. However, the initial CTA did not show luminal irregularities despite extensive infarcts (Fig. 1). Indeed, others have reported normal luminal angiographies of aVA-C4 during the course of the disease [4]. In patient 2, no luminal narrowing or luminal irregularities were detected at any time. The only finding was a subtle dilatation at the site of suspected vascular injury. In patient 1, only T2w sequences were available, which did not show abnormal findings. In patient 2, a subtle, focal T1w hyperintensity of the vessel wall without thickening was detected. In particular, an intramural hematoma was absent. In this respect, findings were not typical for a VA dissection. Nonetheless based on suspected focal extracranial dissection, treatment with anticoagulants for six month over antiplatelet therapy was chosen due to the recurrent multiple embolic infarcts under antiplatelets prior to surgery.

It is notable that the average size of the infarcts in patient 2 was very small (Table 1). Thus, small appositional thrombi might have formed below the detection limit of luminal angiography. Another hypothesis could be that compression in a specific prolonged head position e.g. during sleeping results in thrombus formation. Indeed, Haertel et al. used a nightly application of a stiff neck to treat Bow Hunter Syndrome.

To our knowledge, this is the first report of a patient treated surgically that did not show luminal abnormalities in angiography or VA compression on dynamic imaging. We performed a combined approach of laryngoplasty and partial resection of the C5 transverse process that prevented further strokes.

## Conclusion

The extraforaminal course of an aberrant VA entering at the C3 to C5 level sets the vessels at risk for external compression. Specifically, this compression occurs anterior to the transverse processes, where the vessel is in a fixed and unprotected position. The mechanism of VA compression can be dynamic and thus easily missed on static imaging, such as CTA. A normal luminal angiography does not exclude VA injury. The MR imaging findings can be very subtle and differ from classical VA dissection. Moreover, in contrast to classical VA dissection that is unlikely to reoccur after conservative treatment, recurrent infarcts are typical in aVA-C4 compression. In a patient with recurrent posterior circulation infarcts despite medical management and an aberrant VA, decompressive surgery may be considered even in the absence of verifiable vertebral dissection or compression.
